# Hematological Malignancies: Molecular Mechanisms and Therapy

**DOI:** 10.3390/ijms26062438

**Published:** 2025-03-09

**Authors:** Stella Bouziana

**Affiliations:** 1 Comprehensive Cancer Centre, School of Cancer and Pharmaceutical Medicine, King’s College London, London SE5 8AF, UK; styliani.bouziana@nhs.net; 2 Department of Haematology, King’s College Hospital National Health Service (NHS) Foundation Trust, London SE5 9RS, UK

Hematological malignancies comprise a wide range of relatively rare cancers with a diverse spectrum of biological and clinical presentations. Hematological malignancies can affect both children and adults, with some of them showing a preference for developing at a certain age group [1]. Over the recent decades, the advent and implementation of novel technologies, such as next-generation sequencing (NGS), have enabled deciphering the molecular and genetic signature of hematological malignancies. In addition, epigenetic, transcriptional, and translational analyses have also contributed to delineating the pathogenesis of these cancers [2,3]. Analysis at the single-cell level, including spatial immunophenotyping and multiomics, has resulted in the generation of bulk data, which have significantly contributed, with the aid of bioinformatic pipelines, to gaining a deeper understanding of disease mechanisms in terms of carcinogenesis, disease progression, and resistance to treatment modalities [4,5]. The acquisition of this emerging data has resulted in the development of novel diagnostic tools that offer an accurate and early detection of hematological cancers. In addition, the exploitation of novel technologies, imaging techniques, and the detection of circulating tumor DNA has massively streamlined disease monitoring over natural evolution and treatment of hematological malignancies while offering new predicting, preventive, and prognostic platforms [6,7].

More importantly, the unravelling of molecular pathways has resulted in unprecedented advances in establishing new therapeutic targets and developing innovative types of therapies for hematological cancers. These therapies include targeted therapies, such as bruton’s tyrosine kinase, B-cell lymphoma 2, tyrosine kinase, and isocitrate dehydrogenase inhibitors; immune checkpoint inhibitors that block checkpoint proteins, such as programmed death-1 and programmed death-ligand 1; and a wide variety of immunotherapies, such as monoclonal antibodies and antibody drug conjugates. In addition, epigenetic modifiers, such as DNA methyltransferase inhibitors, have notably improved the overall prognosis of patients with myeloid neoplasms [8]. A major milestone towards curing hematological malignancies has been the advent of allogeneic hematopoietic stem cell transplantation (HSCT), the refinement of which has profoundly improved patient outcomes while minimizing treatment-related toxicities [9]. Another rapidly growing field includes bispecific antibodies, which are engineered to bind to two different targets simultaneously, enhancing the immune system’s ability to attack cancer cells [10]. The landscape of treatment for hematological malignancies is rapidly evolving, with cellular and gene therapies, such as chimeric antigen receptor (CAR) T-cell therapies, constituting groundbreaking options in the treatment armamentarium [8,11]. A combinatorial approach to these novel therapies has revolutionized the field of hemato-oncology, offering long-term remission or even curing hematological patients, even those with highly aggressive and refractory malignancies. 

However, despite this massive progress, there are still hematological malignancies that remain uncured, while there are treatment regimens that confer detrimental or even life-threatening toxicities. Aggressive and refractory types of hematological malignancies entail devastating outcomes, and their successful management to control the disease and induce remission represents a great challenge. Significant research is still needed to be undertaken in order to demystify key pathways towards developing more potent and safer treatments.

This Special Issue aims to compile the latest research advances on investigating the molecular, genetic, and immunological pathways that contribute to the pathogenesis of hematological malignancies or can serve as predictive, preventive, and prognostic disease markers. In addition, this Special Issue focused on collecting cutting-edge knowledge on novel therapeutics of hematological malignancies. Twenty-one manuscripts were submitted for consideration for this Special Issue, and all of them underwent a rigorous peer review process. Finally, eleven articles were accepted for publication and were included in this Special Issue: eight research articles, one review article, one opinion article, and one case report. An overview of the contributed articles is listed below:

1. An experimental *C. elegans* model with homozygous mutations in calreticulin similar to those found in patients with myeloproliferative neoplasms and without the influence of JAK/STAT activation explored the relationship of phenotypic characteristics with transcriptomic alterations. It was shown for the first time that phenotypes related to the alteration of the extracellular matrix, fat levels, and fertility could be a consequence of a partial loss of calreticulin function, suggesting potential targets for innovative therapies by further investigating these effects (Guijarro-Hernández, A.; Hurtado, C.; Urizar-Compains, E.; Ezcurra, B.; Galiana-Sáenz, A.; Baquero, E.; Cabello, J.; Vizmanos, J.L. Myeloproliferative Neoplasm-like Mutations of Calreticulin Induce Phenotypes Associated with Calreticulin Dysfunction in *C. elegans*. *Int. J. Mol. Sci.* 2024, 25, 11606. https://doi.org/10.3390/ijms252111606).

2. An observational cohort aimed to elucidate the impact of immune checkpoint blockade on hematopoietic myeloid clonal expansion in patients with melanoma and non-small cell lung cancer. Sequencing of a targeted panel of genes was performed on serial blood samples to identify mutations, and preliminary results revealed clonal expansion in epigenetic modifier genes, such as *DNMT3A* and *TET2*, potentially indicating a selective pressure of immune checkpoint blockade/T-cell activating therapies on hematopoietic stem cells. Further investigations could assess the predictive role of these mutations as biomarkers of response to immune checkpoint blockade or risk for developing myeloid malignancies (Singh, A.; Trinchant, N.M.; Mishra, R.; Arora, K.; Mehta, S.; Kuzmanovic, T.; Zokaei Nikoo, M.; Singh, I.; Przespolewski, A.C.; Swaminathan, M.; et al. Immune Checkpoint Inhibitor Therapy and Associations with Clonal Hematopoiesis. *Int. J. Mol. Sci.* 2024, 25, 11049. https://doi.org/10.3390/ijms252011049).

3. In patients with de novo T-cell acute lymphoblastic leukemia (ALL), biallelic deletions of genes were investigated by chromosomal microarray analysis. Biallelic loss of 7q34 (*TRB*) and 9p21.3 (*CDKN2A/2B*) was identified in a significant number of patients, while both biallelic deletions were considered to serve as favorable prognostic factors (Risinskaya, N.; Abdulpatakhov, A.; Chabaeva, Y.; Aleshina, O.; Gladysheva, M.; Nikulina, E.; Bolshakov, I.; Yushkova, A.; Dubova, O.; Vasileva, A.; et al. Biallelic Loss of 7q34 (TRB) and 9p21.3 (CDKN2A/2B) in Adult Ph-Negative Acute T-Lymphoblastic Leukemia. *Int. J. Mol. Sci.* 2024, 25, 10482. https://doi.org/10.3390/ijms251910482).

4. In vitro studies in normal and ALL cell lines assessed the impact of the products of a mycovirus-containing *Aspergillus flavus* in certain transcription factors, such as PAX5, Ikaros, and NF-κB. The results showed alterations in the cell cycle and transcription factors along with induced cell apoptosis, highlighting a potential contributing role of environmental factors in leukemogenesis (Tebbi, C.K.; Yan, J.; Sahakian, E.; Mediavilla-Varela, M.; Pinilla-Ibarz, J.; Patel, S.; Rottinghaus, G.E.; Liu, R.Y.; Dennison, C. Mycovirus-Containing Aspergillus flavus Alters Transcription Factors in Normal and Acute Lymphoblastic Leukemia Cells. *Int. J. Mol. Sci.* 2024, 25, 10361. https://doi.org/10.3390/ijms251910361).

5. The role of the *CALR* 52 bp deletion and 5 bp insertion in the pathogenesis of myeloproliferative neoplasms (MPNs) was assessed in vitro in *CALR*-mutated cells offering a deeper understanding of molecular mechanisms in *CALR*-mutated MPNs. It was found that the JAK/STAT and PI3K/Akt/mTOR pathways are activated in *CALR*-mutated cells independent of the CALR and MPL interactions, while *CALR* mutations that impair calreticulin function lead to DNA damage by reducing responses to oxidative stress. (Vadeikienė, R.; Jakštys, B.; Laukaitienė, D.; Šatkauskas, S.; Juozaitytė, E.; Ugenskienė, R. The Role of Mutated Calreticulin in the Pathogenesis of BCR-ABL1-Negative Myeloproliferative Neoplasms. *Int. J. Mol. Sci.* 2024, 25, 9873. https://doi.org/10.3390/ijms25189873).

6. Liquid biopsy studies analyzing plasma free circulating tumor DNA, bone marrow cells, and plasmacytomas for STR profiles in patients with multiple myeloma highlighted the anatomical tumor heterogeneity. In addition, rare *KRAS* or *NRAS* gene mutations were related to a variety of adverse laboratory and clinical factors, suggesting a potential role in predicting an unfavorable prognosis in high-risk multiple myeloma. (Soloveva, M.; Solovev, M.; Risinskaya, N.; Nikulina, E.; Yakutik, I.; Biderman, B.; Obukhova, T.; Chabaeva, Y.; Kulikov, S.; Sudarikov, A.; et al. Loss of Heterozygosity and Mutations in the RAS-ERK Pathway Genes in Tumor Cells of Various Loci in Multiple Myeloma. *Int. J. Mol. Sci.* 2024, 25, 9426. https://doi.org/10.3390/ijms25179426).

7. In vitro studies in Philadelphia-chromosome-positive cell models explored an array of peptide-based inhibitors of CXC chemokine receptor 4 (CXCR4), which seems crucial for the survival of BCR–ABL1-transformed mouse pre-B-cells. The results of this study showed that JM#170, an optimized derivative of EPI-X4, which is an endogenous peptide antagonist of CXCR4, induced cell death both in mouse and human cell lines by activating the intrinsic apoptotic pathway. This killing effect was stronger when Imatinib, an ABL1 kinase inhibitor, was combined with JM#170 which may represent a novel drug against Philadelphia positive B-ALL (Pohl, J.; Litz, A.; El Ayoubi, O.; Rodríguez-Alfonso, A.; Ständker, L.; Harms, M.; Münch, J.; Jumaa, H.; Datta, M. An Optimized Peptide Antagonist of CXCR4 Limits Survival of BCR–ABL1-Transformed Cells in Philadelphia-Chromosome-Positive B-Cell Acute Lymphoblastic Leukemia. *Int. J. Mol. Sci.* 2024, 25, 8306. https://doi.org/10.3390/ijms25158306).

8. Metabolic and lipidomic analysis using liquid chromatography/mass spectrometry set to differentiate four T-ALL cell lines belonging to the same TAL/LMO genetic subgroup based on metabolic profiles. Significant differences and similarities were found in metabolites across the four cell lines, while bioinformatic tools identified differences in enzymes involved in diverse pathways among the cell lines (Alabed, H.B.R.; Pellegrino, R.M.; Buratta, S.; Lema Fernandez, A.G.; La Starza, R.; Urbanelli, L.; Mecucci, C.; Emiliani, C.; Gorello, P. Metabolic Profiling as an Approach to Differentiate T-Cell Acute Lymphoblastic Leukemia Cell Lines Belonging to the Same Genetic Subgroup. *Int. J. Mol. Sci.* 2024, 25, 3921. https://doi.org/10.3390/ijms25073921).

9. The most recent advances in haploidentical allogeneic HSCT for treating pediatric hematological disorders were reviewed. Outcomes of haploidentical allogeneic HSCT have significantly improved; however, multiple challenges are still in place needing further research to be tackled (Marszołek, A.; Leśniak, M.; Sekunda, A.; Siwek, A.; Skiba, Z.; Lejman, M.; Zawitkowska, J. Haploidentical HSCT in the Treatment of Pediatric Hematological Disorders. *Int. J. Mol. Sci.* 2024, 25, 6380. https://doi.org/10.3390/ijms25126380).

10. An opinion article presented the current landscape of secondary malignancies post CAR T-cell therapies given the new safety concern triggered by the Food and Drug Administration announcement in November 2023 on reported cases of T-cell malignancies in patients previously treated with either anti-CD19 or anti-BCMA autologous CAR T-cell therapies. A proposed strategy for future research aiming at potentially diminishing or abrogating the risk of developing secondary malignancies after CAR T-cell therapies was also presented (Bouziana, S.; Bouzianas, D. The Current Landscape of Secondary Malignancies after CAR T-Cell Therapies: How Could Malignancies Be Prevented? *Int. J. Mol. Sci.* 2024, 25, 9518. https://doi.org/10.3390/ijms25179518).

11. An interesting case report of a patient with an *ETV6::ABL1*-positive myeloid neoplasm was presented. The diagnosis was established by NGS, which identified both *ETV6::ABL1* type A and B fusion transcripts and was later confirmed by FISH, while the patient was treated with imatinib mesylate, achieving a deep, durable response lasting more than one year according to the last follow-up (Bochicchio, M.T.; Marconi, G.; Baldazzi, C.; Bandini, L.; Ruggieri, F.; Lucchesi, A.; Agostinelli, C.; Sabattini, E.; Orsatti, A.; Ferrari, A.; et al. ETV6::ABL1-Positive Myeloid Neoplasm: A Case of a Durable Response to Imatinib Mesylate without Additional or Previous Treatment. *Int. J. Mol. Sci.* 2024, 25, 118. https://doi.org/10.3390/ijms25010118).

This compilation of articles encompasses a diverse spectrum of research ranging from early stage preliminary pre-clinical studies to clinical cohorts. All of these articles tried to shed light into key molecular, genetic and cellular pathways of different hematological malignancies in an attempt to better understand the pathogenesis, discover new biomarkers and develop novel therapies. Further research is warranted to expand and validate these results in more complex in vivo models and subsequent clinical studies. This highlights the need for collaboration of researchers from a multi-disciplinary background in an effort to further accelerate advances in the field of hematological malignancies with an overall aim of translating research findings into clinical practice and improving patient outcomes. We hope that this Special Issue will serve towards this direction by attracting the scientific interest of researchers across the globe.

## References

[1] Siegel R.L., Miller K.D., Fuchs H.E., Jemal A. (2021). Cancer statistics, 2021. CA Cancer J. Clin..

[2] Kwon R., Yeung C.C. (2024). Advances in next-generation sequencing and emerging technologies for hematologic malignancies. Haematologica.

[3] Cao X., Huber S., Ahari A.J., Traube F.R., Seifert M., Oakes C.C., Secheyko P., Vilov S., Scheller I.F., Wagner N. (2024). Analysis of 3760 hematologic malignancies reveals rare transcriptomic aberrations of driver genes. Genome Med..

[4] Campillo-Marcos I., Alvarez-Errico D., Alandes R.A., Mereu E., Esteller M. (2021). Single-cell technologies and analyses in hematopoiesis and hematological malignancies. Exp. Hematol..

[5] Larsson L., Frisén J., Lundeberg J. (2021). Spatially resolved transcriptomics adds a new dimension to genomics. Nat. Methods.

[6] Talotta D., Almasri M., Cosentino C., Gaidano G., Moia R. (2023). Liquid biopsy in hematological malignancies: Current and future applications. Front. Oncol..

[7] Parghane R.V., Basu S. (2024). Role of Novel Quantitative Imaging Techniques in Hematological Malignancies. PET Clin..

[8] Lica J.J., Pradhan B., Safi K., Jakóbkiewicz-Banecka J., Hellmann A. (2024). Promising Therapeutic Strategies for Hematologic Malignancies: Innovations and Potential. Molecules.

[9] Phelan R., Chen M., Bupp C., Bolon Y.-T., Broglie L., Brunner-Grady J., Burns L.J., Chhabra S., Christianson D., Cusatis R. (2022). Updated Trends in Hematopoietic Cell Transplantation in the United States with an Additional Focus on Adolescent and Young Adult Transplantation Activity and Outcomes. Transplant. Cell. Ther..

[10] Goebeler M.-E., Bargou R.C. (2020). T cell-engaging therapies—BiTEs and beyond. Nat. Rev. Clin. Oncol..

[11] Cappell K.M., Kochenderfer J.N. (2023). Long-term outcomes following CAR T cell therapy: What we know so far. Nat. Rev. Clin. Oncol..

